# Color and Translucency Compatibility Among Various Resin-Based Composites and Layering Strategies

**DOI:** 10.3390/dj13040173

**Published:** 2025-04-18

**Authors:** Elena Bianca Varvara, Cristina Gasparik, Javier Ruiz-López, Alexandra Iulia Aghiorghiesei, Bogdan Culic, Diana Dudea

**Affiliations:** 1 Department of Prosthetic Dentistry and Dental Materials, Iuliu Hațieganu University of Medicine and Pharmacy, 400006 Cluj-Napoca, Romania; bianca.varvara@umfcluj.ro (E.B.V.); irimie.alexandra@umfcluj.ro (A.I.A.); bculic@umfcluj.ro (B.C.); ddudea@umfcluj.ro (D.D.); 2 Department of Optics, Faculty of Science, Campus de Fuente Nueva, Edificio Mecenas, University of Granada, ibs-Granada, 18071 Granada, Spain

**Keywords:** color, translucency, resin composites, composite layering, biomimetic dentistry

## Abstract

(1) **Background:** Natural-looking dental restorations require careful selection of the restorative material, with color and translucency characteristics similar to the natural dental structures. (2) **Objectives:** This research aimed to evaluate if there is compatibility regarding the color and translucency between different commercial RBCs in different layering recipes. (3) **Methods**: Sixty 1 mm thick disk specimens were produced from three different RBCs: ESS (Essentia-GC), BEG (Brilliant Ever Glow-Coltene), and IPS (IPS Empress Direct-Ivoclar Vivadent). Three different opacities and translucencies (enamel, dentin, and opaque shades) from each system were used in four recipes (R1-enamel, R2-dentin, R3-enamel and dentin, R4-enamel, dentin, and opaque) to obtain single-, double-, and triple-layered samples, respecting the anatomical layering technique. CIE L*, a*, b*, C*, h^0^ coordinates were recorded, and the relative translucency parameter (RTP_00_) was calculated. Further, the color differences (ΔE_00_) and the difference in translucencies ΔRTP_00_ were analyzed between the materials and between the layered recipes. (4) **Results**: The CIE L* and h° color coordinates and RTP_00_ showed significant differences among all three RBCs for all four recipes (*p* < 0.001). The decreasing order of translucency for each recipe was R1: ESS > BEG > IPS, R2 and R3: BEG > IPS > ESS, R4: BEG > ESS > IPS. Important differences were found in color and translucency among the recipes for each of the three RBCs tested (*p* < 0.001). The decreasing order of translucency for the tested RBCs was ESS: R1 > R3 > R4 > R2, BEG and IPS: R1 > R3 > R2 > R4. (5) **Conclusions:** No significant compatibility was observed in color and translucency among different layering recipes of the same composite materials nor between similar layering recipes when using different composites. The color differences between materials were more significant than the differences in translucency for each recipe.

## 1. Introduction

Resin-based composites (RBCs) are a widely used category of dental materials that are highly compatible with additive, minimally invasive dentistry [1]. Their improved physical and mechanical properties, cost-effectiveness, and repairability make them suitable restorative materials for small to medium cavities or cosmetic bonded veneers when addressing the shape and structural imperfections of the dental structure [2,3]. Clinically, resin composite restorations’ natural and esthetically pleasing outcomes are primarily linked to their optical integration with the surrounding tooth structure [4,5,6,7].

An RBC’s intrinsic esthetic qualities are influenced by its color and translucency [2,8]. The color of an RBC is typically produced by pigments or, more recently, by specific configurations of fillers engineered to blend seamlessly with the surroundings [2,9,10].

In color research, the colorimetric assessment employs the CIE L*, a*, b* color coordinates based on the CIELAB color space (Commission Internationale de l’Éclairage) [11]. Variations in the color coordinates, especially along the CIE a* (red-green) axis and the CIE b* (yellow-blue) axis, result in noticeable changes in the perceived and measured hue and intensity of colors. Additionally, the color’s lightness (CIE L*) affects brightness, giving colors a lighter or, conversely, a darker appearance. Using color difference formulas (CIELAB − ΔE^*^_ab_; CIEDE2000 − ΔE_00_) established by the CIE further enhances the clinical significance of color assessment in dentistry, specifically from a clinical perspective, through their comparison with their respective visual thresholds for color differences in dentistry [12,13]. A color match is considered acceptable if the color difference falls below the acceptability threshold (AT) and is considered excellent if this value is below the perceptibility threshold (PT) [13].

The CIELAB color coordinates can be recorded using color measurement instruments. The Spectroshade Micro instrument (Medical High Technologies, Milan, Italy) is a non-contact imaging spectrophotometer commonly utilized in clinical and laboratory studies [14,15,16]. A recent study demonstrated the validity and reliability of this instrument for in vitro dental research [14]. The authors concluded that the differences between the Spectroshade Micro and a spectroradiometer (the gold standard for color measurement) were clinically insignificant, suggesting that the dental spectrophotometer can be effectively used for clinical shade matching and in vitro dental color research.

Translucency is an essential optical property that allows light to pass through the restoration–tooth complex, resulting in a mixed appearance between the restorative material and the dental substrate. When clinically evaluating a restoration’s translucency, it is determined whether it resembles human enamel and if the human eye can detect the differences between the dental structure and the restorative portion [17].

Similarly to natural dental tissues, RBCs display various degrees of translucency and opacity for the enamel and dentin layers intended for replacement. The classification of RBCs, based on their optical properties, primarily relates to the translucency level of the material in comparison to dental structures, with terminology differing among various brands. By altering the composition, more opaque materials have been developed to mask a dyschromic substrate (opaque, opaque dentin, or dentine). Meanwhile, body shades (body, dentin) have been developed to mimic the mass of dentin. The enamel layers are replaced with more translucent composites (enamel); increments of incisal or translucent composites are recommended for a more detailed characterization of opalescent zones [18].

Translucency is evaluated by the translucency parameter (TP), which is calculated as the color difference of a sample over ideal black and ideal white backgrounds [19]. Since ideal backgrounds are only used for theoretical calculations, the relative translucency parameter (RTP) is actually used with the scale corresponding to the color difference between the existing backgrounds [20]. To emphasize the clinical relevance, the difference in translucency must be compared with the respective perceptibility and acceptability thresholds [21].

The RBC color codification varies from the VITA shade designations to terms suggesting tooth age (e.g., young enamel) [22]. However, studies report inconsistencies in color among RBC shades with similar designations [23,24]. In addition, although the RBC classification based on composition is still in use, a newer classification that takes into account the number of available shades is more clinically oriented: single-shaded (one-shaded), which claims to match all sixteen VITA classical shades; group-shaded (cloud shade) comprising fewer RBC syringes that match specific VITA classical shades; and multi-shaded RBCs, which are the traditional systems with multiple syringes that correspond to each VITA classical shade [3].

In practice, factors such as the position and size of the restoration, the practitioner’s skills and preferences, and the patient’s concerns about appearance guide the selection of restorative protocols. These can range from using a single-layer, single-shade RBCs (either enamel or body) to complex layering with various shades and opacities [3,25]. Over time, several layering concepts were developed based on an improved understanding of the histo-anatomy of the tooth and its light interaction with natural dental tissues: the bi-laminar approach, anatomical stratification technique [26], penta-laminar approach [27], and natural layering technique [28]. The evolution of methods used for direct restorations with resin composites was driven by the same aim of enhanced reliability and clinical simplification [28], employing a single layer of dentin and a defined enamel thickness, which serves as a straightforward and reproducible procedure for creating anterior restorations [29,30].

Multilayered resin composites with varying translucencies and opacities can enhance the integration of the restoration [31]; however, when selecting a complex layering strategy, a good understanding of the optical characteristics of dental substrate is vital to achieving biomimetic restorations [32,33,34].

Several studies have analyzed the color of single-layered versus multilayered restorations [35,36,37,38,39]; still, the clinical implications of using three layers of composite of different brands of modern RBCs in reduced thickness have been less studied. This in vitro study aimed to compare the color and translucency of layering recipes across various RBCs and to assess different layering recipes for the same RBC. The tested hypotheses were that (1) the layering recipe did not affect the color coordinates and translucency parameters of the RBCs, and (2) there was no significant difference in the color coordinates and translucency parameters among the various layering recipes for the same RBC.

## 2. Materials and Methods

### 2.1. Material Selection and Sample Preparation

Three RBCs were selected for this study: Essentia (GC Europe, Leuven, Belgium)—ESS, Brilliant Ever Glow (Coltene, Altstätten, Switzerland)—BEG, and IPS Empress Direct (Ivoclar Vivadent, Schaan, Liechtenstein)—IPS (Table 1).

**Figure 1:**
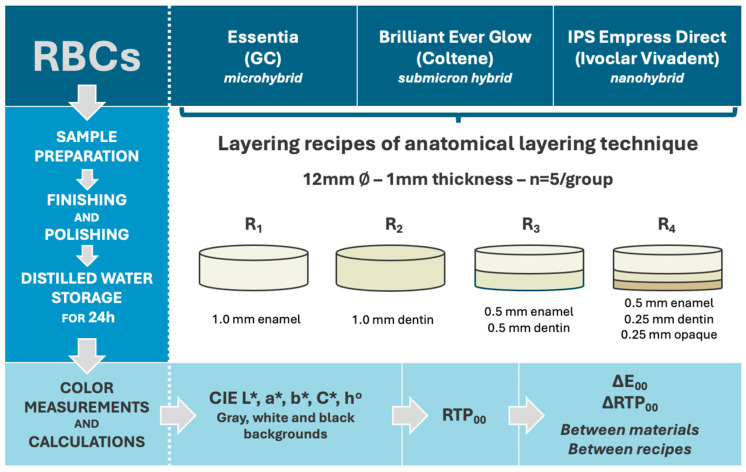
The flow diagram of the study.

Enamel, body/dentin, and opaque shades from each system were utilized using four strategies to produce single-, double-, and triple-layered samples, following the anatomical layering technique. A flow diagram of the study is presented in Figure 1.

A total of 60 samples (n = 5) [40,41,42] with 1.0 mm thickness were fabricated using four different recipes (R1, R2, R3, and R4). Single-layer samples were fabricated using only enamel (R1) or dentin (R2) shade. Double-layer samples (R3) were obtained from 0.5 mm of dentin and 0.5 mm of enamel shade, while triple-layered samples (R4) were fabricated by layering 0.25 mm of opaque, 0.25 mm of dentin, and 0.5 mm of enamel shade (Table 2).

The samples were obtained using a stainless-steel mold (Porcelain Sampler, Smile Line, St-Imier, Switzerland). After packing the resin composite into the mold, a transparent mylar strip and a glass slab were pressed over the resin to create a smooth surface and ensure a uniform thickness of the disk specimens. All samples were light-cured for 20 s on each side, according to the manufacturer’s recommendations with a light-curing unit that produced a power of 1800 mW/cm^2^ (Led.H Orto, Woodpecker, Guilin, China).

Double-layered samples (1.0 mm thickness) were acquired from a pre-polymerized 0.5 mm dentin sample and a 0.5 mm enamel shade increment that was added to the mold after the base of the mold was lowered to facilitate the placement of a 0.5 mm enamel shade increment. Similarly, triple-layer samples were made using three different increments of 0.25 mm opaque, 0.25 mm dentin, and 0.5 mm enamel, with light curing between each sample.

All the samples were polished manually on one surface with sandpaper (P800, P1500, P2000, P5000). The thickness of each sample was verified with a digital caliper (Z22855, Milomex Ltd., Pulloxhill, UK), and only specimens with a thickness of 1.0 mm (±0.05 mm) were included in the study. Afterward, the samples were cleaned using distilled water in an ultrasound bath for 5 min, placed individually in plastic zip-lock bags filled with 5 mL of distilled water to ensure the complete immersion of the samples, and stored at room temperature (25 °C) in a dark container.

The specimens were divided into 12 groups (n = 5) according to the restoration system and the resin-composite shade combination used for the layering technique. Following the fabrication process, all specimens were kept hydrated in distilled water for 24 h before all measurements.

### 2.2. Color Measurement and Calculation

The CIE L*, a*, b*, C*, and h° color coordinates of the samples were measured using a non-contact dental spectrophotometer (SpectroShade Micro, Medical High Technologies, Milan, Italy) with a 45°/0° illumination and measuring geometry and using CIE 2° Standard Observer and the CIE D65 Standard Illuminant. The mouthpiece of the spectrophotometer was positioned perpendicular to a custom-built stand, ensuring precise repositioning of the instrument and isolation from ambient light. Calibration was performed as per the manufacturer’s instructions between each sample. A sucrose solution (refractive index n = 1.453) was used as a coupling agent to maintain optical continuity between the specimens and the standardized backgrounds [43]. For each specimen, three consecutive measurements, without replacement, were taken. A single trained operator conducted all measurements.

For color measurement, all samples were assessed against a standardized gray background (L* = 50.63, a* = 1.74, b* = −3.32), and the color differences between 1. the samples fabricated using the same recipe, in different materials and 2. the samples built from the same material, but using different recipes, were calculated as follows:

$${∆E_{00}=\left[{\left(\frac{∆L^{\prime }}{k_{L}S_{L}}\right)}^{2}+{\left(\frac{∆C^{\prime }}{k_{C}S_{C}}\right)}^{2}+{\left(\frac{∆H^{\prime }}{k_{H}S_{H}}\right)}^{2}+R_{T}\left(\frac{∆C^{\prime }}{k_{C}S_{C}}\right)\left(\frac{∆H^{\prime }}{k_{H}S_{H}}\right)\right]}^{\frac{1}{2}}$$

where ΔL′, ΔC′, and ΔH′ are the differences in lightness, chroma, and hue, respectively, for the same crown measured over two different substrates. The parametric factors k_L_, k_C_, and k_H_ are correction terms for experimental conditions and were set to 1 in the present study. S_L_, S_C_, and S_H_ refer to the weighting functions that adjust the total color difference considering the location variation in the color difference pair in L′, a′, and b′ coordinates. Finally, the parameter R_T_ is the rotation function, which accounts for the interaction between chroma and hue differences in the blue region [11,44].

The color differences calculated were compared to the 50:50% visual thresholds for perceptibility (PT_00_) and acceptability (AT_00_) [13,45]: PT_00_ = 0.8 and AT_00_ = 1.8. The contribution of lightness (ΔL_00_), chroma (ΔC_00_), and hue (ΔH_00_) to the total color difference (ΔE_00_) was calculated using the following equations [46]:

$$∆L_{00}=\frac{∆L^{\prime }}{k_{L}S_{L}};∆C_{00}=\frac{∆C^{\prime }}{k_{C}S_{C}};∆H_{00}=\frac{∆H^{\prime }}{k_{H}S_{H}}$$

The translucency of the samples was assessed using the relative translucency parameter (RTP_00_), which represents the difference between the CIE L*, a*, b* color coordinates of each sample on black (B) (L* = 2.13, a* = 2.58, and b* = −3.57) and white (W) (L* = 89.93, a* = 0.61, b* = −4.42) backgrounds [11,19,44]:

$${{RTP}_{00}=\left[{\left(\frac{{L\prime }_{B}-{L\prime }_{W}}{k_{L}S_{L}}\right)}^{2}+{\left(\frac{{C\prime }_{B}-{C\prime }_{W}}{k_{C}S_{C}}\right)}^{2}+{\left(\frac{{H\prime }_{B}-{H\prime }_{W}}{k_{H}S_{H}}\right)}^{2}+R_{T}\left(\frac{{C\prime }_{B}-{C\prime }_{W}}{k_{C}S_{C}}\right)\left(\frac{{H\prime }_{B}-{H\prime }_{W}}{k_{H}S_{H}}\right)\right]}^{\frac{1}{2}}$$

Differences in translucency (ΔRTP_00_) were interpreted according to their respective visual thresholds (TPT_00_ = 2.6 and TAT_00_ = 0.6, respectively) [12,21].

### 2.3. Statistical Analysis

Levene’s test and the Shapiro–Wilk test were conducted (α = 0.05) to assess the normality of the data. Equal variances and a normal distribution could not be assumed for all groups and parameters. Consequently, the Kruskal–Wallis one-way analysis of variance by ranks was employed to evaluate the differences in the CIE L*, a*, b*, C*, h°, and RTP_00_. Multiple comparisons were analyzed using the Mann–Whitney U test with a Bonferroni correction (α = 0.001). The statistical analysis was performed using standard software (SPSS Statistics 20.0.0, IBM, Armonk, NY, USA).

## 3. Results

The mean values and standard deviations of CIE L*, a*, b*, C*, h°, and RTP_00_ by material and recipe are presented in Table 3.

### 3.1. Comparison of the Same Layering Recipes Across Different Materials

The CIE L* and h° color coordinates and RTP_00_ showed significant differences among the three RBCs for all four recipes (*p* < 0.001). The CIE a*, b*, and C* color coordinates significantly differed among the tested RBCs, except for the comparison between BEG and IPS in R1 and R2 recipes (*p* ≥ 0.001).

**Figure 2:**
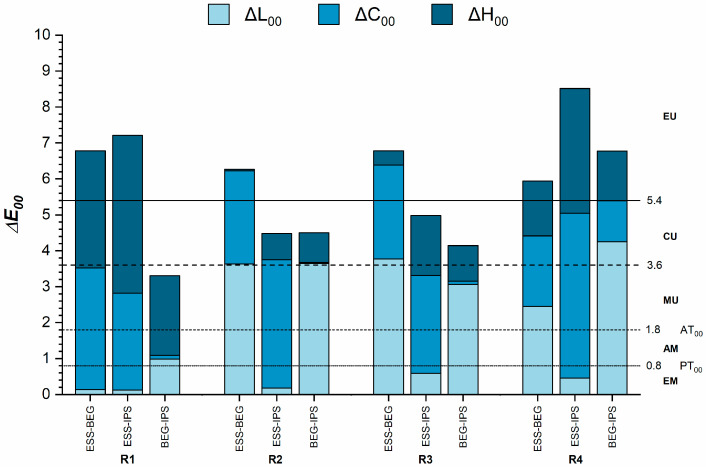
Color differences between different materials for the same recipes. ΔE_00_, color difference; ΔL_00_, lightness difference; ΔC_00_, chroma difference; ΔH_00_, hue difference; AM, acceptable match; AT_00_, acceptability threshold; CU, clearly unacceptable; EM, excellent match; EU, extremely unacceptable; MU, moderately unacceptable; PT_00_, perceptibility threshold.

Figure 2 presents the color differences among the RBCs for each recipe. In all comparisons, the color differences exceeded the AT_00_. The smallest color differences were noted in the R1 recipe between BEG and IPS (ΔE_00_ = 3.3), while the largest differences occurred for the R4 recipe between ESS and IPS (ΔE_00_ = 8.5). Overall, the R2 and R3 recipes exhibited almost similar color differences between materials, with differences exceeding two (clearly unacceptable match) or three times (extremely unacceptable match) the AT_00_. In the case of the R1 and R4 recipes, the color differences between the RBCs were extremely unacceptable, except between BEG and IPS for the R1 recipe, which had moderately unacceptable color differences.

For the R1 recipe (enamel only), the differences in color among the materials were mainly due to variations in chroma and hue. The decreasing order of the CIE C* coordinate (chroma) was BEG > IPS > ESS, while the decreasing order for the h° coordinate (hue) was ESS > BEG > IPS (Table 3).

In the R2 recipe (dentin only), the observed color differences were associated with variations in lightness and chroma. The CIE L* coordinate (lightness) varied in the following order: ESS > IPS > BEG. For the CIE C* coordinate, the order was IPS > BEG > ESS (Table 3).

For the R3 and R4 recipes (layered recipes), the color differences were also affected by variations in lightness and chroma. In the case of the R3 recipe, the decreasing order of the CIE L* coordinate was ESS > IPS > BEG, while for the CIE C* coordinate, it was BEG > IPS > ESS. For the R4 recipe, the CIE L* coordinate varied as follows: IPS > ESS > BEG, and the CIE C* coordinate was IPS > BEG > ESS (Table 3).

**Figure 3:**
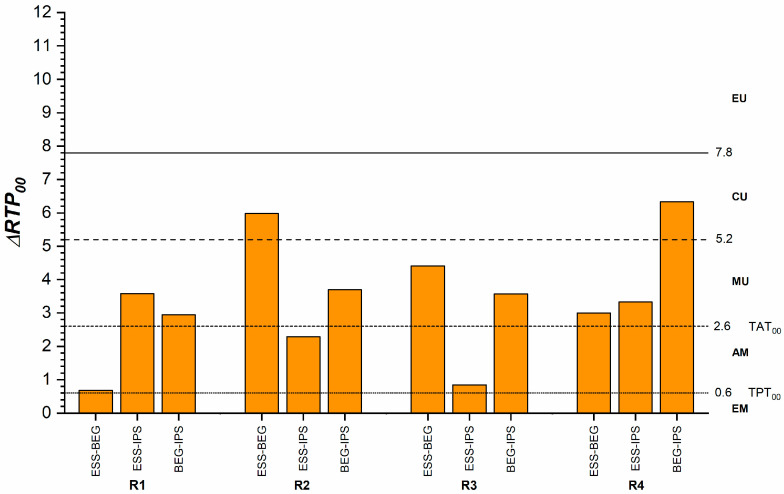
Translucency differences between different materials for the same recipes. ΔRTP_00_, relative translucency parameter difference; AM, acceptable match; CU, clearly unacceptable; EM, excellent match; EU, extremely unacceptable; MU, moderately unacceptable; TAT_00_, acceptability threshold; TPT_00_, perceptibility threshold.

Figure 3 illustrates differences in translucency among the RBCs for each recipe. The smallest differences, close to the TPT_00_, were observed in the R1 recipe between ESS and BEG and the R3 recipe between ESS and IPS. The largest differences were noted for the R2 recipe between ESS and BEG, as well as for the R4 recipe between BEG and IPS. In these cases, the differences were unacceptable (>2 times the TAT_00_). The decreasing order of translucency for each recipe was R1: ESS > BEG > IPS, R2 and R3: BEG > IPS > ESS, R4: BEG > ESS > IPS.

### 3.2. Comparison of the Different Layering Recipes Within the Same Materials

For ESS, the CIE b* and C* color coordinates and the RTP_00_ significantly differed among recipes (*p* < 0.001). The CIE L* and h° color coordinates were significantly different between the recipes, except for the R3 and R4 comparisons. The CIE a* coordinate of the R2 recipe differed significantly from the other recipes (*p* < 0.001). The R4 recipe was the most chromatic among the recipes, regardless of the material.

For BEG, the CIE L* coordinate showed no significant differences between R1 and R3 or between R2 and R4 recipes, while the CIE a* coordinate exhibited no significant differences in any of the comparisons (*p* ≥ 0.001). The CIE C* and h° coordinates were significantly different across the recipes, except in the comparison between R2 and R3. The RTP_00_ differed significantly in all recipe comparisons (*p* < 0.001).

For IPS, the CIE L*, b*, and C* coordinates, as well as RTP_00_, showed significant differences across the recipes (*p* < 0.001). The CIE a* and h° coordinates significantly differed across recipes, except for the comparisons between R1 and R4 and R2 and R3 (*p* < 0.001).

**Figure 4:**
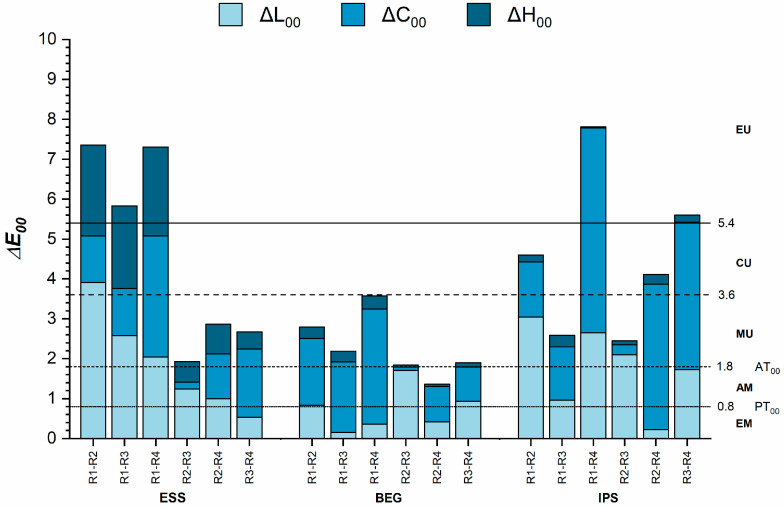
Color differences between different recipes for the same materials. ΔE_00_, color difference; ΔL_00_, lightness difference; ΔC_00_, chroma difference; ΔH_00_, hue difference; AM, acceptable match; AT_00_, acceptability threshold; CU, clearly unacceptable; EM, excellent match; EU, extremely unacceptable; MU, moderately unacceptable; PT_00_, perceptibility threshold.

Figure 4 shows the color differences among the recipes for each of the tested RBCs. For ESS, the smallest color differences were found between the R2 and R3 recipes, where the values were at the level of the AT_00_. In the case of BEG, the smallest color differences were observed between the R2 and R4 recipes, with values below the AT_00_. For IPS, the color differences were highly above the AT_00_; however, the smallest differences were noted between the R1 and R3 recipes, as well as between the R2 and R3 recipes.

For ESS and IPS, the color differences between R1 (enamel) and R2 (dentin) were attributed to differences in lightness, whereas for BEG, they were due to variations in chroma. In the cases of ESS and IPS, the dentin appeared lighter than the enamel (CIE L* coordinate R2 > R1), while for BEG, the dentin was more chromatic than the enamel (CIE C* coordinate R2 > R1) (Table 3).

For ESS and IPS, the R2 recipe (dentin) appeared lighter than the R3 recipe (enamel and dentin), even though the chroma and hue were almost identical for both materials. This also clarifies that the primary contribution to the color difference between the R2 and R3 recipes is due to the variation in lightness.

**Figure 5:**
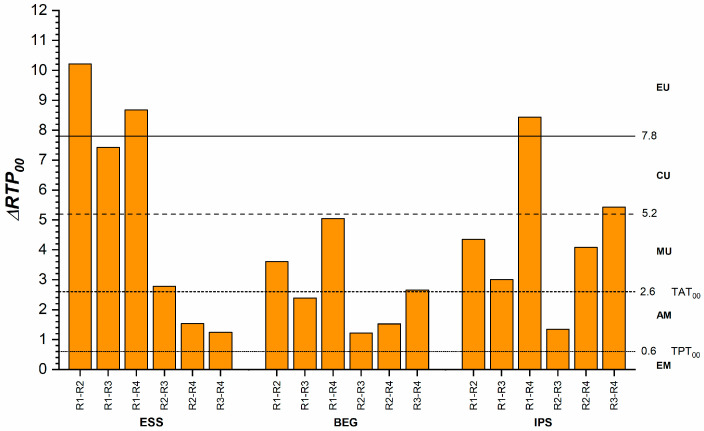
Translucency differences between different recipes for the same materials. ΔRTP_00_, relative translucency parameter difference; AM, acceptable match; CU, clearly unacceptable; EM, excellent match; EU, extremely unacceptable; MU, moderately unacceptable; TAT_00_, acceptability threshold; TPT_00_, perceptibility threshold.

Translucency differences among various recipes for the same materials are shown in Figure 5. For ESS, the smallest differences were identified between the R2 and R4 recipes, as well as between R3 and R4, with values below the TAT_00_. For BEG, the smallest differences were observed between R2 and R3 and between R2 and R4, also below the TAT_00_. In the case of IPS, translucency differences were below the TAT_00_ only between R2 and R3. The decreasing order of translucency for the three RBCs was ESS: R1 > R3 > R4 > R2, BEG and IPS: R1 > R3 > R2 > R4 (Table 3).

## 4. Discussion

When placing a minimally invasive restoration using RBCs, the clinician must understand the colorimetrical and optical behavior of the materials and apply the appropriate technique to maximize the potential of the composite. Therefore, knowing the differences between various combinations of enamel, dentin, and opaque materials can be a practical aid in selecting one layering recipe over another.

The study aimed to evaluate the color and relative translucency parameters of both enamel and dentin samples, as well as to determine the influence of the layered recipes on the sample outcomes. The layered samples were constructed with a thickness of 1 mm to simulate a minimally invasive direct veneer restoration. The total thickness of 1 mm was divided into various configurations, with a predetermined thickness of 0.5 mm allocated for the final enamel layer, corresponding to the average thickness of natural buccal enamel. Furthermore, the study sought to assess the impact of an opaque shade at a thickness of 0.25 mm on the overall color and translucency of the restoration; therefore, the triple-layered samples were created with 0.5 mm enamel, 0.25 mm dentin, and 0.25 mm opaque.

The first hypothesis was rejected because significant differences were found in the color and translucency of the three tested RBCs for each of the four recipes (*p* < 0.001). ESS enamel exhibited the highest translucency among the enamel-only samples (R1), whereas IPS enamel displayed the lowest. This finding aligns with another study [4], which reported that Essentia Light Enamel samples had a greater translucency parameter than Empress Direct A2 Enamel. Moreover, in this study, the enamel samples fabricated from ESS and BEG exceeded the TP_00_ value of 16.8 reported recently for 1.0 mm natural enamel slabs [47], while the translucency of IPS was slightly below. However, even considering the trend, the exact comparison of the values is limited due to the differences in the measurement setup and instruments used.

In RBCs, translucency relates to the nature, size, shape, and density of the fillers, as well as the monomeric composition and the differences in the refractive index among the composite’s components. Translucency in a resin composite increases when the refractive index of the resin matrix closely matches that of the filler as reported in previous studies [4,48], so the increased translucency of the ESS enamel samples can be attributed to the Bis-GMA content (RI = 1.54), which has a refractive index comparable to that of the silica filler (RI = 1.53). Furthermore, although the filler content is higher (81 wt%, 65 vol%), implying increased light dispersion, the nanometric dimensions of the pre-polymerized fillers (10 nm), barium glass (300 nm), and fumed silica (16 nm) may explain its increased translucency. BEG enamel samples have an almost similar filler content by weight (79%) and volume (64%), but the inorganic filler size, reaching 1500 nm, has a lower translucency, as reported by the manufacturer [49]. However, the differences in translucency found in this study were close to the perceptibility threshold, which signifies an almost excellent match between the two materials (Figure 3). For the IPS enamel, the filler (barium glass filler, mixed oxide, Ba-Al-fluorosilicate glass) and the addition of pigments might be responsible for the increased lightness and higher opacity.

Additionally, for the R1 recipe (enamel samples), BEG was the most chromatic, followed by IPS and ESS. ESS showed a greenish hue, despite having low chroma, while BEG and IPS appeared more yellowish with slightly higher chroma. The color differences between the enamel shades (ESS vs. IPS and ESS vs. BEG), resulting from variations in hue and chroma that exceed the AT_00_, account for designating IPS and BEG as chromatic materials (high chroma), whereas ESS is categorized as “achromatic” by the manufacturer (low chroma).

In general, there is no consensus among commercial brands on the designation of translucency and opacity levels of RBCs. Designating “artificial enamel” resins is difficult, as each variation displays specific individualized properties [18]. IPS is an artificial chromatic enamel keyed to the Vita shade guide. Due to its increased chroma and hue, which resemble the A1 hues characteristic of young teeth, it can provide a chromatic basis for the restoration. Utilizing it as milky-white semi-translucent enamel for creating halos or high-value areas with white effects would also be an excellent choice. BEG yields an intermediate outcome, with increased chroma, but with higher translucency and lower value; the Bleach Translucent, which was not tested in this study, might be characterized by higher value. With its high translucency and low chroma, ESS may best provide translucency, particularly to deep areas along the incisal and proximal edges. Increasing its thickness could make the grayish effect more noticeable compared to BEG and IPS. The lowest chroma of the ESS is characteristic of the “achromatic artificial enamel”.

This study aligns with previous research indicating that the shade and brand of resin composite affect translucency values [25,50]. Although we included similar shades of enamel and dentine, we observed color variations between the commercial brands, as well as differences in translucency for both enamel and dentine.

The R2 recipe in this study aimed to represent the opacities intended to replace the dentin; however, the RTP_00_ values for all materials exceeded the values corresponding to a natural dentin thickness of 1.0 mm, as reported by Paravina et al. [47] (RTP_00_= 4.20). Once again, this variation can largely be attributed to differences in the measurement setup and instruments.

ESS’s most opaque dentin shade may be considered a technical solution to complement the increased translucency observed in the corresponding enamel. This was also true for the hue; the more yellowish hue of ESS dentin differed from that of ESS enamel shades (more greenish) and was closer to the values reported for the dentin of the other two systems. In the case of dentin shades, the color differences were primarily due to variations in lightness and chroma, with hue remaining more consistent. ESS exhibited the highest lightness, consistent with its low translucency, while BEG showed the lowest; in both instances, this can be attributed to the level of translucency.

For the R3 recipe (with an equal thickness of dentin and enamel layers), BEG exhibited the most increased translucency. Still, it was also the most chromatic, showing an intermediary translucency between the enamel and dentin samples. In contrast, the translucency for the R4 recipe, which included a third layer of opaque composite, was consistently lower than that of the R3 recipe with only two layers, enamel and dentin. Interestingly, for the ESS case, the translucency of the tri-layered samples exceeded that of the dentin (single layer), whereas for BEG and IPS, it was lower. This result may be partly attributed to the very low translucency of the dentin in the ESS case.

The second hypothesis was also rejected because important differences were found in color and translucency among the recipes for each of the three RBCs tested (*p* < 0.001). The RTP_00_ varied significantly (*p* < 0.001) among the recipes for all materials; as expected, in every instance, the RTP_00_ for the enamel (R1) was greater than that for R2 (dentine). Moreover, the RTP_00_ of R1 was greater than that of R3 (two-layered) and R4 (three-layered) for every material.

R4, the three-layer recipe, was the least translucent in every instance, demonstrating the lowest RTP_00_ for IPS. Additionally, the difference in translucency between R3 and R4 was most pronounced for IPS, suggesting that at a thickness of 0.25 mm, the opaque composite has the most significant opacifying effect among the other opaque versions.

Regarding the color attributes, the color differences between R1 (enamel) and R2 (dentin) were mainly due to differences in lightness for IPS and ESS, while for BEG, they were due to differences in chroma. For all materials, the dentin was lighter and more chromatic than the enamel. The chroma of R4—when all three opacities were used for layering—was the greatest for all materials, followed by R2, R3, and R1. This might also explain why chroma contributed most notably to the color differences calculated between the R1–R4, R2–R4, and R3–R4 recipes. Similar results were reported in a previous study by La Rosa et al. [38] that investigated the color of layered enamel and dentin samples fabricated from Clearfil Majesty ES-2 Premium, Estelite Asteria, and Brilliant Everglow. The authors concluded that in all layering recipes where enamel was replaced by dentin, unacceptable color differences were observed, which resulted from the increase in lightness and chroma.

The R3 recipe (enamel and dentin) proved to be less chromatic than R2 (dentin only), which suggests that when enamel is superimposed over dentine of the same thickness, the chroma will decrease. The lightness of all materials was greatest for the dentine samples (R2) compared to the other ESS and BEG recipes, suggesting that any addition to these will make the final result be perceived as darker. IPS exhibited the highest lightness in R4 due to the addition of the opaque layer to the three-layer recipe.

When considering the layering technique in a minimally invasive restoration, the clinical situation must be judged individually, considering not only the translucency and color attributes of the components used but also the optical effect generated by their combination [37]. Our study aligns with other research highlighting the importance of maintaining a proper balance between dentin and enamel layer thickness to achieve an optimal shade [37,38]. Additionally, the thickness and shade of the more translucent enamel layer can influence the overall shade of the dentin [51,52]. In this research, this situation was particularly relevant to ESS. Similar results were reported in another study, which found that the optical properties of the cover material generally had a more significant influence on the color appearance of the layered samples than the underlying material [36,37].

For ESS, associating enamel with dentin (as in the R3 recipe) or with both dentin and opaque (as in the R4 recipe) makes the translucency and color changes of the complex results unacceptable compared to the enamel (R1 vs. R2, R1 vs. R3, R1 vs. R4); however, even in R4, the translucency remains above the dentine’s values. This may be due to the strong effect of the increased translucency of the enamel, even at 0.5 mm thickness. Conversely, the translucency and the color of the dentin are less affected by the presence of the enamel or the opaque layer (R2 vs. R3 and R2 vs. R4), with both recipes leading to a moderately unacceptable color difference from the dentin layer. The ΔRTP_00_ and the color difference between the bi- and triple-layer recipes (R2 vs. R3) remain also moderately unacceptable. This suggests that a layering strategy in a minimally invasive restoration instead of enamel alone will strongly influence ESS’s original translucency and color. In addition, the underlying dentine will increase the lightness, which is beneficial for young teeth, mainly because the artificial enamel is, in this case, high in lightness and low in chroma. Furthermore, adding the opaque layer will not highly modify the final optical outcome in its tested thickness (0.25 mm).

For IPS, there was less variation from the original translucency of the enamel (R1 vs. R3), or dentine (R2 vs. R3) in the two-layer formula, probably due to the lower translucency of the chromatic enamel. The only layer showing a large variation in translucency compared with the other recipes is R4, which shows the opaque layer’s strong effect. In addition, the color differences between layers ranged from moderately acceptable to moderately unacceptable, except for the difference between R4 and the other layers. The differences between enamel and dentin (R1 vs. R2) were mainly due to variations in lightness, likely because the pigments used for coloring belonged to the same color family. In this case, using only enamel for minimally invasive restorations might be a simpler and equally suitable option than layering enamel and dentin. However, the color of the opaque layer strongly influences the color of the enamel, dentin, and even the enamel–dentin complex. All these color differences arise from variations in lightness or chroma. Using the opaque in the layering protocol will likely lead to important changes compared to using only enamel and dentin.

The smallest differences between the recipes were recorded for BEG, with minimal values between R1-R3 and R2-R3 falling below the TAT_00_. This indicates that the combination of dentin and enamel of equal thickness will not influence the initial translucency of the enamel or dentin layers when considered individually. However, the addition of an opaque layer will have a considerable effect on the translucency of both the enamel and dentine. The color differences between the recipes were less important, staying within the acceptable and moderately unacceptable range, and were primarily influenced by chroma. These findings suggest that layering methods using this system will yield a less different outcome than using only enamel or dentin.

This research aligns with Khashayar et al. [35], who tested the natural layering protocol using composite resins categorized into two concepts: (1) dentin and enamel share the same shade but differ in translucency, and (2) dentin and enamel exhibit different shades, with enamel being highly translucent. They found that the composites in the first concept were less sensitive to layer thickness, resulting in a more predictable shade. In contrast, small variations in thickness in the enamel layer of second concept composites significantly impact the final color of the restoration. In our case, ESS, with its highly translucent enamel, resembles the composites from the second concept. Conversely, IPS, which has chromatic enamel and dentin sharing the same color designation, falls under the first concept. However, when opaque material was added to the layering in IPS in R4, both color and translucency were strongly affected.

Clinicians should adjust their material selection according to the color and translucency characteristics of each RBC system. Single-shade composites may perform well for smaller restorations or whiter teeth but might not completely replicate the natural tooth color in more complex cases. Additionally, group shade composites tend to perform better on lighter substrates, while darker substrates may need customized solutions. For larger restorations or instances requiring precise color matching, clinicians should consider using composite systems with multiple shades and translucencies, as translucency significantly affects the final color of a restoration. More translucent materials enable the underlying substrate to influence the visible shade, which can either enhance or hinder color matching depending on the color of the substrate.

One limitation of the study is that only three RBC brands were tested, and the samples had a flat surface. Moreover, this study’s results only apply to the A1/B1 shade and 1.0 mm total thickness. The behavior of other shades, particularly darker or more translucent ones, may differ from those investigated in the present study. Additionally, instead of a bench-top spectrophotometer, a non-contact clinical spectrophotometer was utilized to record the color coordinates of the samples, simulating in vivo color determination. Another limitation is that the samples were tested against a neutral gray background instead of using tooth-colored substrates.

Further research is necessary to evaluate the effect of tooth-colored backgrounds and the masking ability of the proposed recipes. Future studies should consider testing the color and translucency of various layering recipes on contoured samples that better simulate clinical conditions. Also, future studies simulating UV exposure and artificial aging will bring invaluable findings on the long-term color stability of novel RBCs.

## 5. Conclusions

Within the limitations of the study, the following was concluded:

1. The color differences between materials were more significant than the differences in translucency for each recipe.

2. The most important differences in color and translucency between the recipes were identified for ESS and IPS. These differences were primarily attributed to the significant variations in color and translucency between enamel and dentin.

3. A layering strategy in a minimally invasive restoration, rather than using enamel alone, will significantly influence the original translucency and color for ESS.

4. Layering methods using BEG or IPS result in a less different outcome than using only enamel or dentin. However, important modifications are expected when adding the opaque layer.

## Figures and Tables

**Table 1:** Composition of the resin-based composites.

Material/Shade Designation/Codification	Type	Shade/Opacity	Composition	Batch Number
**Essentia** (GC, Tokyo, Japan) Group-shade **Non-VITA shade** **designation** **Codification:** ESS	Microhybrid	LE (light enamel)	Matrix: UDMA, Bis-MEPP, Bis-EMA, Bis-GMA, TEGDMA Filler: Pre-polymerized fillers (10 nm), barium glass (300 nm), fumed silica (16 nm), 81 wt% (65%)	180130A
		LD (light dentin)	Matrix: UDMA, Bis-MEPP, Bis-EMA, Bis-GMA, TEGDMA Prepolymerised fillers (17 μm): strontium glass (400 nm), barium glass (300 nm), lanthanide flüoride (100 nm), fumed silica (16 nm) FAISi glass (850 nm), 76–81 wt %	170905A
		ML (masking liner)	UDMA (10–25%), homogeneously dispersed ultra-fine fillers titanium dioxide (0.5–1%), silane, reaction products with silica (0.5–1%), diphenyl (2,4,6-trimethylbenzoyl) phosphine oxide (0.5–1%) SiO2: Diameter particle structure = 2.5–50 nm Diameter agglomerate = 5–50 mm	170816A
**Brilliant Ever Glow** (Coltene) Group-shade VITA shade designation Codification: BEG	Universal submicron hybrid composite	Translucent	Not available	I36341
		A1/B1	Matrix: Bis-GMA (1–5 wt%), Bis-EMA (10–15 wt%), TEGDMA (1–5 wt%) Fillers: zinc oxide (<1.5%), ytterbium trifluoride (<10%), (submicron) barium glass fillers, dental glass and nanosilica; pre-polymerised fillers, filler content by weight 79%; filler content by volume 64%, inorganic filler size 20–1500 nm	H27017
		OA1	Not available	I37878
**IPS Empress Direct** (Ivoclar Vivadent, Schaan, Lichtenstein) Multishade VITA shade designation Codification: IPS	Nanohybrid	A1E (enamel, shade A1)	Matrix: UDMA (10–25%), TCDDA (2.5–10%) Fillers:Ba-Al-fluorosilicate glass, barium glass filler, mixed oxide ytterbium trifluoride, prepolymer, mixed oxide and glass particles 75–79 wt%, 52–59 vol	W95078
		A1D (dentin, shade A1)	Monomers: Bis-GMA, UDMA (10–25%), and TCDDA (2.5–10%) Fillers: Ba-Al-fluorosilicate glass (50.2 wt%), ytterbium trifluoride (9.8 wt%), mixed oxide, prepolymer (19.6 wt%)	Y07490
		Opaque	Matrix: UDMA (10–25%), DDDMA (10–25%) Dimethacrylates (53.9 wt. %) Fillers: Barium glass, ytterbium trifluoride (2.5–10%), Al-fluorosilicate glass, spheroid mixed oxide (43.4 wt %). Total content of inorganic fillers (23.2 vol %). Particle size 0.04–3.0 μm, mean particle size: 0.7 μm	W98712

The information was gathered from the manufacturer’s websites, product brochures, and references [4,40]. **Abbreviations:** Bis-EMA, ethoxylated bisphenol-A dimethacrylate; Bis-GMA, bisphenol A-glycidyl methacrylate; RBC, resin-based composite; TCDDA, tricyclodecane dimethanol dimethacrylate; TEGDMA, triethylene glycol dimethacrylate; UDMA, urethane dimethacrylate; DDDMA, decanediol dimethacrylate.

**Table 2:** Detailing of the recipes.

Recipes	ESS	BEG	IPS
R1	1 mm LE	1 mm T	1 mm A1 E
R2	1 mm LD	1 mm A1/B1	1 mm A1 D
R3	0.5 mm LD + LE	0.5 mm A1/B1 + 0.5 mm T	0.5 mm A1 D + 0.5 mm A1 E
R4	0.25 mm ML + 0.25 mm LD + 0.5 mm LE	0.25 mm OA1 + 0.25 mm A1/B1 + 0.5 mm T	0.25 mm Opaque + 0.25 mm A1D + 0.5 mm A1E

**Table 3:** Mean values and standard deviations of CIE L*, a*, b*, C*, h° chromatic coordinates, and RTP_00_ by material and recipe.

Material	Recipe	L*	a*	b*	C*	h°	RTP_00_
ESS	R1	68.06 (0.30)	−1.55 (0.39) ^bE^	0.03 (0.19)	1.56 (0.40)	178.30 (6.40)	19.19 (0.36)
	R2	75.12 (0.54)	−0.92 (0.20) ^C^	5.53 (0.31)	5.61 (0.32)	99.41 (1.96)	8.98 (0.25)
	R3	73.09 (0.92) ^A^	−1.45 (0.43) ^aCE^	4.79 (0.15)	5.02 (0.20)	106.72 (4.62)^A^	11.76 (0.28)
	R4	72.93 (1.58) ^A^	−2.41 (1.09) ^cE^	7.03 (0.21)	7.49 (0.49)	108.50 (7.48) ^A^	10.52 (0.52)
BEG	R1	66.99 (0.49) ^C^	−1.49 (0.35) ^bD^	7.88 (0.39) ^b^	8.03 (0.36) ^b^	100.73 (2.73) ^BC^	18.56 (0.36)
	R2	68.81 (0.64) ^B^	−1.44 (0.05) ^D^	11.07 (0.46) ^aA^	11.16 (0.46) ^aA^	97.45 (0.50) ^BC^	14.96 (0.56)
	R3	66.58 (0.32) ^C^	−1.55 (0.13) ^aD^	10.77 (0.36) ^A^	10.88 (0.35) ^A^	98.22 (0.83) ^B^	16.17 (0.38)
	R4	68.17 (0.74) ^B^	−1.50 (0.12) ^cD^	12.74 (0.37)	12.83 (0.36)	96.70 (0.65) ^C^	13.52 (0.92)
IPS	R1	69.20 (0.18)	0.48 (0.15) ^A^	7.63 (0.43) ^b^	7.65 (0.44) ^b^	86.43 (0.95) ^E^	15.61 (0.35)
	R2	74.13 (0.55)	−0.01 (0.30) ^B^	11.21 (0.36) ^a^	11.21 (0.36) ^a^	90.29 (0.93) ^D^	11.27 (0.22)
	R3	71.12 (0.89)	−0.02 (0.27) ^B^	10.20 (0.22)	10.21 (0.23)	90.08 (1.51) ^D^	12.61 (0.21)
	R4	75.24 (0.70)	0.85 (0.37) ^A^	17.54 (0.84)	17.56 (0.85)	87.25 (1.14) ^E^	7.18 (0.73)

Comparison of the same recipes by different materials: the same lowercase letter in superscript within the same column indicates no significant statistical difference (*p* ≥ 0.001). Comparison of different recipes within the same material: the same uppercase letter in superscript within the same column indicates no significant statistical difference (*p* ≥ 0.001).

## Data Availability

The data presented in this study are available on request from the corresponding author.
